# Special issue on lagomorph disease investigations

**DOI:** 10.1177/10406387241267942

**Published:** 2024-09-12

**Authors:** Javier Asin, Fábio Abade dos Santos, Denise M. Imai

**Affiliations:** Department of Pathology, Microbiology, and Immunology, University of California–Davis, Davis, CA, USA; California Animal Health and Food Safety Laboratory, San Bernardino Branch, University of California–Davis, Davis, CA, USA; National Institute for Agrarian and Veterinary Research (INIAV), Oeiras, Portugal and Faculty of Veterinary Medicine, Lusofona University, Lisboa, Portugal; Department of Pathology, Microbiology, and Immunology, University of California–Davis, Davis, CA, USA; Comparative Pathology Laboratory, University of California–Davis, Davis, CA, USA


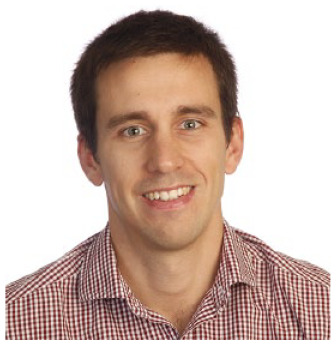



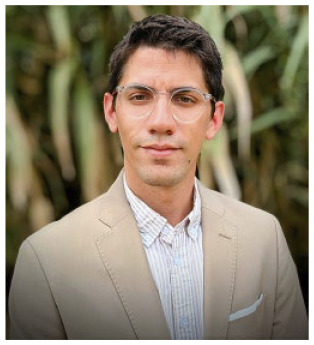



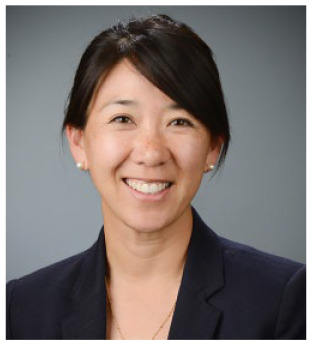


Lagomorphs are mammals in the order *Lagomorpha*, which is divided into 2 families: *Ochotonidae* and *Leporidae*.^15^ The former includes several species of pikas (genus *Ochotona*), which are small, short-limbed lagomorphs with short round ears native to Asia and North America. Leporids are larger, have long ears and hindlimbs, and indigenous species originate from all continents except Antarctica and Oceania, although invasive species exist in the latter. The *Leporidae* family includes hares and jackrabbits (genus *Lepus*), and various species of rabbits (genera *Brachylagus*, *Bunolagus*, *Caprolagus*, *Nesolagus*, *Oryctolagus*, *Pentalagus*, *Poelagus*, *Pronolagus*, *Romerolagus*, *Sylvilagus*). The European rabbit (*Oryctolagus cuniculus*) is possibly the best-known lagomorph due to its domestication and common use as a pet, as well as for research purposes and meat and fur production.

Interest in lagomorph science has grown in the last few years. In this special issue of JVDI, we compile articles on lagomorph pathology and laboratory testing that we hope can help veterinary laboratory diagnosticians, pathologists, and other veterinarians when dealing with these species.

In the United States, the American Veterinary Medical Association estimated that 1.2% of households (1.5 million households) owned at least one pet rabbit in 2021, and rabbits were considered among the 5 most common non-dog or -cat pets in the country.^3^ While rabbit meat production and consumption are currently not as popular in the United States as in Europe or China,^22^ small U.S. commercial and hobbyist producers that breed rabbits for meat and other purposes are common. Owners, producers, and veterinary practitioners are increasingly interested in diseases that affect rabbits, with samples for detection of infectious agents, sera, biopsies, and autopsies being submitted to diagnostic laboratories worldwide. This rising interest in the diseases of domesticated rabbits is highlighted by the multiple retrospective studies published in this special issue from the western United States, Hong Kong, eastern Canada, the United Kingdom, and the Spanish Canary Islands off the west coast of Africa.^7,8,12,13,23^ The global spectrum of diseases is similar; neoplastic diseases of the female reproductive tract and integumentary system predominate, and bacterial abscesses are common.^7,8,12,13^ However, there are geographic variabilities in infectious, non-infectious, and neoplastic diseases,^7,8,12,13^ as well as specific patterns of disease prevalence determined by the purpose of use of the domesticated rabbit.^12^ These retrospective studies emphasize the diagnostic utility of the clinical history, including the purpose of use and geographic origin, histologic evaluation,^13^ and ancillary testing, such as conventional microbiology. Interestingly, almost half of the bacterial isolates obtained from pet rabbits in a diagnostic laboratory in Hong Kong were multidrug-resistant.^23^

Some wild lagomorphs are very important components of ecosystems and trophic chains as they serve as a protein source for predators.^6^ Others are restricted to certain geographic locations and considered vulnerable, threatened, or critically endangered.^1^ There are very few studies on the pathology and causes of morbidity and mortality of native North American lagomorphs. In this special issue, we included retrospective studies of diseases and pathology of cottontail rabbits (*Sylvilagus* spp.) in the eastern United States and American pikas (*Ochotona princeps*), which are among the first of their kind in the scientific literature.^4,21^ The emergence and global spread of rabbit hemorrhagic disease virus 2 (*Caliciviridae*, *Lagovirus europaeus* GI.2) and the increasing list of leporid species susceptible to this virus have fueled concerns about animals in this taxon, especially from a conservation perspective.^1,2^ In fact, there have been a few attempts to vaccinate small populations of endangered wild rabbits such as the riparian brush rabbit (*Sylvilagus bachmani riparius*) in California,^11^ and novel laboratory tests for this disease are being developed and adapted.^2,20^ Endoparasitoses are common in rabbits and hares, and pathology is an excellent diagnostic tool for some of these diseases.^9^

Other articles included in this issue are a comprehensive review of mycotoxicoses in rabbits^17^; a retrospective study of cases of a classic rabbit disease, *Clostridium spiroforme*–associated enteric disease^18^; a histologic characterization of catecholamine-induced cardiomyopathy in laboratory New Zealand white rabbits^14^; and several case reports of unique disease presentations.^5,10,16,19^
